# *Carex parva* and *Carex scabrirostris* adopt diverse response strategies to adapt to low-light conditions

**DOI:** 10.3389/fpls.2024.1432539

**Published:** 2024-10-14

**Authors:** Wanting Liu, Rong Fan, Siyu Yang, Sibo Chen, Yulin Huang, Wenli Ji

**Affiliations:** College of Landscape Architecture and Art, Northwest A&F University, Xianyang, China

**Keywords:** *Carex*, low-light environment, photosynthesis, ecological strategy, dauciform root

## Abstract

**Introduction:**

In recent years, the visible light intensity of lawns has significantly decreased due to obstructions caused by urban shading objects. *Carex* has a competitive advantage over other turfgrass in low-light conditions and extensive management. Therefore, exploring their survival strategy in low-light environments is of great significance.

**Methods:**

This study focuses on two species of *Carex*, *Carex parva* and *Carex scabrirostris*, and investigates their response to low-light conditions (150 μmol/m^2^/s) by simulating urban lawn conditions. Biomass allocation characteristics, leaf anatomical features, biochemical parameters, root morphology and photosynthetic parameters were measured.

**Results:**

(a) Peroxidase activity, specific leaf area, and relative water content are key factors influencing the photosynthetic capacity of the two *Carex* species. (b) Under low-light conditions, photosynthetic parameters, leaf physiological indicators, and biomass allocation of the two *Carex* species were significantly affected (*p*<0.05). Both *Carex* species increased their investment in leaf biomass, maintained lateral root growth, and cleared reactive oxygen species to maintain their physiological balance. (c) In the simulated urban low-light environment, neither *C. parva* nor *C. scabrirostris* produced dauciform roots.

**Discussion:**

In terms of response strategies, *C. scabrirostris* is a high-photosynthesis investing species with high productivity under low-light conditions, whereas *C. parva* exhibits minimal response, indicating a slow investment. *C. scabrirostris* has greater potential for application in low-light environments compared to *C. parva*. These results provide a theoretical basis for the cultivation and application of these two *Carex* species, as well as the expansion of turfgrass germplasm resources.

## 1 Introduction

In recent years, lawns have played an increasingly significant role in landscape greening (Thompson and Kao-Kniffin, 2019), providing not only social benefits and ecosystem services to the urban environment but also substantial economic and ecological benefits (Trammell et al., 2019). Currently, urban buildings, artificial structures, and dense tree canopies have created numerous shaded areas, resulting in a significant reduction in visible light intensity within cities (Francini et al., 2023). However, most types of turfgrass do not adapt well to excessively shaded environments (Zhang et al., 2016). Given that light conditions are an important factor restricting turfgrass growth in urban environments, selecting a shade-tolerant turfgrass species is imperative (Fu et al., 2020).

*Carex*, one of the most ecologically diverse genera (Jakovljević et al., 2014), is currently being used in urban lawns, and their growth status is being explored (Shokoya et al., 2022). Compared with other turfgrasses, the Carex genus has the advantage of growing in low-light or low-maintenance conditions (Więcław, 2017). It has been found that the *Carex* genus produces dauciform roots (Güsewell and Schroth, 2017), which are effective in absorbing water and nutrients from deeper soils and storing large amounts of nutrients in infertile soils, providing a stable source of nutrients for plant growth (Shane et al., 2005). Research has found that the *Carex* genus can produce dauciform roots (Güsewell and Schroth, 2017), which are capable of effectively absorbing water and nutrients from deep soil layers. As a result, dauciform roots can store large amounts of nutrients in infertile soils, providing a stable nutrient source for plant growth. Previous studies have found that *Carex* species with dauciform root systems are more likely to occur in areas with high light intensity and lower phosphorus availability (Playsted et al., 2006; Brundrett, 2009). However, the formation of dauciform roots under low-light conditions in urban lawns is mainly unknown.

Typical responses of plants to low-light environments include an increase in aboveground biomass, thinner leaves, and a larger specific leaf area (SLA) (Milla and Matesanz, 2017). Additionally, low-light conditions reduce the maximum carboxylation capacity (Vc_max_), maximum electron transport rate (J_max_), light saturation point (LSP), and net photosynthetic rate (Pn_max_) (Haque et al., 2017; Fu et al., 2017; Sun et al., 2023). These changes are the result of the long-term coordination between leaf functional traits and photosynthesis (Nam et al., 2017; Wright et al., 2004). Leaf anatomical structure reflects important photosynthetic physiological characteristics (Maza-Villalobos et al., 2022). Wang et al. (2023) studied factors influencing plant photosynthetic capacity based on leaf anatomy. It is important to select *Carex* species that can efficiently utilize light energy, considering that *Carex* species lack palisade tissue in mesophyll cells (Wang et al., 2023).

Plants also adapt to low-light conditions by altering their physiological and metabolic processes (Zhang et al., 2022; Kittipornkul et al., 2023). Specifically, the chlorophyll content decreases while osmoregulatory substances accumulate, and antioxidant enzyme activity increases (Wu, 2016; Wu et al., 2021). In the absence of light, plants secrete many osmoregulatory substances to maintain normal osmotic pressure in the cytoplasm (Huang et al., 2022). Lower light will imbalance the internal scavenging system of reactive oxygen species, leading to membrane lipid peroxidation (Liang et al., 2009; Zhang et al., 2022). Antioxidant enzymes, such as peroxidase (POD), can eliminate reactive oxygen species, maintaining redox homeostasis (Liu et al., 2021).

As a significant concept in ecology, the leaf economics spectrum reveals the coordination and trade-offs among various plant traits (Wright et al., 2004). Plants adopt different investment strategies in low-light environments (Wright et al., 2004). Plants that prioritize rapid return on investment often have larger and thinner leaves, whereas plants employing resource-storage strategies exhibit contrasting characteristics (Jiang et al., 2023). These traits result from the ongoing interactions between plants and their environments over time (Violle et al., 2007). Similarly, the same species may exhibit different structural characteristics in different environments (Hu et al., 2022). Studying the variability and plasticity of plant traits also contributes to understanding their growth strategies under different environmental conditions (Fajardo and Siefert, 2016; Lafont Rapnouil et al., 2023). Additionally, plant functional traits can be categorized into soft traits and hard traits. In order to obtain certain key physiological traits that are difficult to measure in real time in the field, such as indicators related to photosynthesis, other easily observable soft traits can be used as proxies (Cornelissen et al., 2003). Since different plant organs and tissues respond differently to environmental changes (Funk and Cornwell, 2013), it is of significant importance to identify functional trait indicators in *Carex* species that are associated with the efficient utilization of light energy.

*C. parva* is a perennial herb of the genus *Carex* in the family Cyperaceae. It has high resistance to drought and shaded conditions, making it suitable for extensive management. It is also a turfgrass species with excellent potential for urban areas (Dai et al., 2010). *C. scabrirostris*, an endemic species of high research value in China, commonly grows alongside *C. parva* (Dai et al., 2010). Currently, there are limited studies on the environmental adaptability and survival strategies of these two *Carex* species (Wang et al., 2023). Out of 865 species of sedge plants in China, only three, including *C. parva*, have been observed to develop dauciform roots in their natural habitats (Gao and Yang, 2016). Further exploring the excellent potential and application value of *Carex* species in low-light environments and providing parental materials for domesticating and introducing urban lawns in China.

This study aims to investigate the response changes of *C. parva* and *C. scabrirostris* under low-light environments, as well as their different growth strategies in heterogeneous environments. Specifically, we propose and explore the following questions: (a) Can we identify the key factors influencing the photosynthetic capacity of these two species of *Carex*? (b) How do the responses of these two *Carex* species change in simulated low-light urban environments, and how do they adapt to low-light environments? (c) Do these two species of *Carex* have different growth strategies under two different light environments, and which *Carex* has a broader range of potential applications in low-light environments? This study further explores the excellent potential and application value of *Carex* species in low-light environments, providing parental material and preliminary theoretical support for the introduction and domestication of these species in urban lawns in China.

## 2 Materials and methods

### 2.1 Study area

The plant materials used in this experiment were *C. parva* and *C. scabrirostris*, which belong to the genus *Carex* in the family Cyperaceae. They were collected from the Taibai Mountains (34°10’N, 107°58’E), a predominantly high-altitude grassland situated at an elevation of 3,620 m (Dai et al., 2010). The area experiences an annual precipitation of 751.8 mm, with a yearly relative humidity of 70% and an average annual temperature of 11°C (Cao et al., 2016). We selected three sample areas in the Taibai Mountains, with a distance of 20 m between each sample area. Each area was dug up to form three sample clusters measuring 20 cm × 20 cm × 20 cm (length, width, and depth) (Falcioni et al., 2017). Soil samples were taken at the four corners and the central area of the sample plot using the plum 5-point method (Falcioni et al., 2017).

The plant materials were stored in the climate chamber of Northwest A&F University (34°26’N, 108°06’E). The chamber was established under the following conditions: light duration of 14 h day/10 h night, day/night temperature of 25/18°C, light intensity of 150 μmol/m²/s, air relative humidity controlled at 65%–75%, and soil moisture maintained consistent with the original habitat. The parameters were chosen based on the shaded lawn environments in Shaanxi as the main reason (Wang et al., 2023). The experiment was conducted during the optimal growth season for the selected plants, which was late July.

### 2.2 Sampling and measurements

After being collected from the field, some plants were immediately processed upon arrival at the laboratory, while the remaining plants were kept in a growth chamber to simulate a low-light environment for 80 days before processing. Five randomly selected samples of each species were harvested for analysis. During the harvesting process, plant photosynthesis was measured, including the light response curve and the CO_2_ response curve, using the LI-6400XT (LI-COR, Lincoln, NE, USA). Photosynthetically active radiation (PAR) levels were set at 2000, 1500, 1200, 1000, 750, 500, 250, 200, 150, 100, 50, 25, and 0 μmol m^−2^ s^−1^, with a CO_2_ concentration of 400 μmol mol^−1^. The CO_2_ concentration in the sample chamber was varied between 400, 300, 200, 150, 100, and 50 μmol mol^−1^, and then set back to 400, 600, 800, 1000, 1200, 1500, and 2000 μmol mol^−1^, with a constant PAR of 1000 μmol mol^−1^. The hyperbolic tangent model was used to fit indices such as the LSP, light compensation point (LCP), dark respiration rate (RD), maximum carboxylation rate (Vc_max_), and maximum electron transport rate (J_max_) (Wang et al., 2023).

Plant morphological indices were measured next. Leaf area was measured using the LI-3000C portable leaf area meter. Three mature leaves were randomly selected, and their length, average width, maximum width, and leaf area were measured. Using a caliper (De Antonio et al., 2023), the thickness of the leaf on the same side as the main vein was measured, and the fresh weight and dry weight of these leaves were recorded. Additionally, the anatomical and structural characteristics of leaf sections were observed using a MoticBA410 microscope (Jiang et al., 2023). Images were captured, and parameters such as upper epidermis thickness, lower epidermis (LET) thickness, and cuticle thickness (CUT) were documented. Root morphology, including root length, root volume, and root surface area, was measured using the Winrhizo software. Among the 175 specimens of *C. parva* and 76 specimens of *C. scabrirostris* collected in the field, 0 and 64 plants with dauciform roots (lateral roots with swollen axes) were observed, respectively. The aboveground parts of the plants were separated on a quartz surface, and their fresh weight and dry weight were measured individually using an electronic balance.

Last, physiological indices of the plants were measured. Chlorophyll a and b were extracted from fresh leaves using 95% (v/v) ethanol according to Lichtenthaler and Wellburn (1983). Malondialdehyde (MDA) content was determined using the thiobarbituric acid method, proline mass fraction using the sulfosalicylic acid extraction ninhydrin colorimetric method, Soluble protein (SP) content using the Coomassie Brilliant Blue method, and POD content using the guaiacol method (Weng et al., 2015).

### 2.3 Statistical analysis

Data analysis for this study was conducted using SPSS 19.0, and graphs were generated using Origin 22. The normality of all data was assessed using the Shapiro–Wilk test, and the homogeneity of variances was assessed using Levene’s test. In case the results did not follow a normal distribution, a square root transformation was applied to achieve normality. Fisher’s LSD analysis was used to determine the statistical significance of differences between treatments (*p* < 0.05). To assess the variability of plant functional traits between species, the coefficient of variation (CV) was calculated using the formula CV = (SD ÷ M) × 100%, where SD is the standard deviation and M is the mean. Traits with a CV exceeding 50% were considered ecologically adaptive traits, while traits with a lower CV served as indicators of systematic evolution, reflecting species’ adaptive potential (Zhang et al., 2021). Additionally, the plasticity index (PI) was used to characterize the response of two *Carex* species to different environments. It was calculated as PI = (max − min)/max, where max and min represent the maximum and minimum values of a certain trait. Traits with PI > 0.6 were defined as sensitive indicators of habitat response, while traits with PI < 0.2 were considered inert indicators of habitat response. Spearman correlation analysis was used to examine the relationships among plant functional traits. To further screen relevant indicators for the efficient utilization of light in two types of *Carex*, redundancy analysis (RDA) was performed on nine functional trait indicators. Detrended correspondence analysis (DCA) was conducted on RDA data (Grinn-Gofroń et al., 2018). Indicators were selected based on causality and correlation analysis, excluding those with poor response and indicators directly derived from basic indicators (Flexas et al., 2022). RDA was used to explore the associations between photosynthetic traits and plant physiological ecology, ranking the contribution values for each indicator (Liu et al., 2021). Finally, the RDA results were compared and validated with the corresponding correlation analysis to ensure the accuracy of the findings (Dong et al., 2022).

## 3 Result

### 3.1 Changes in photosynthetic parameters

**Figure 1:**
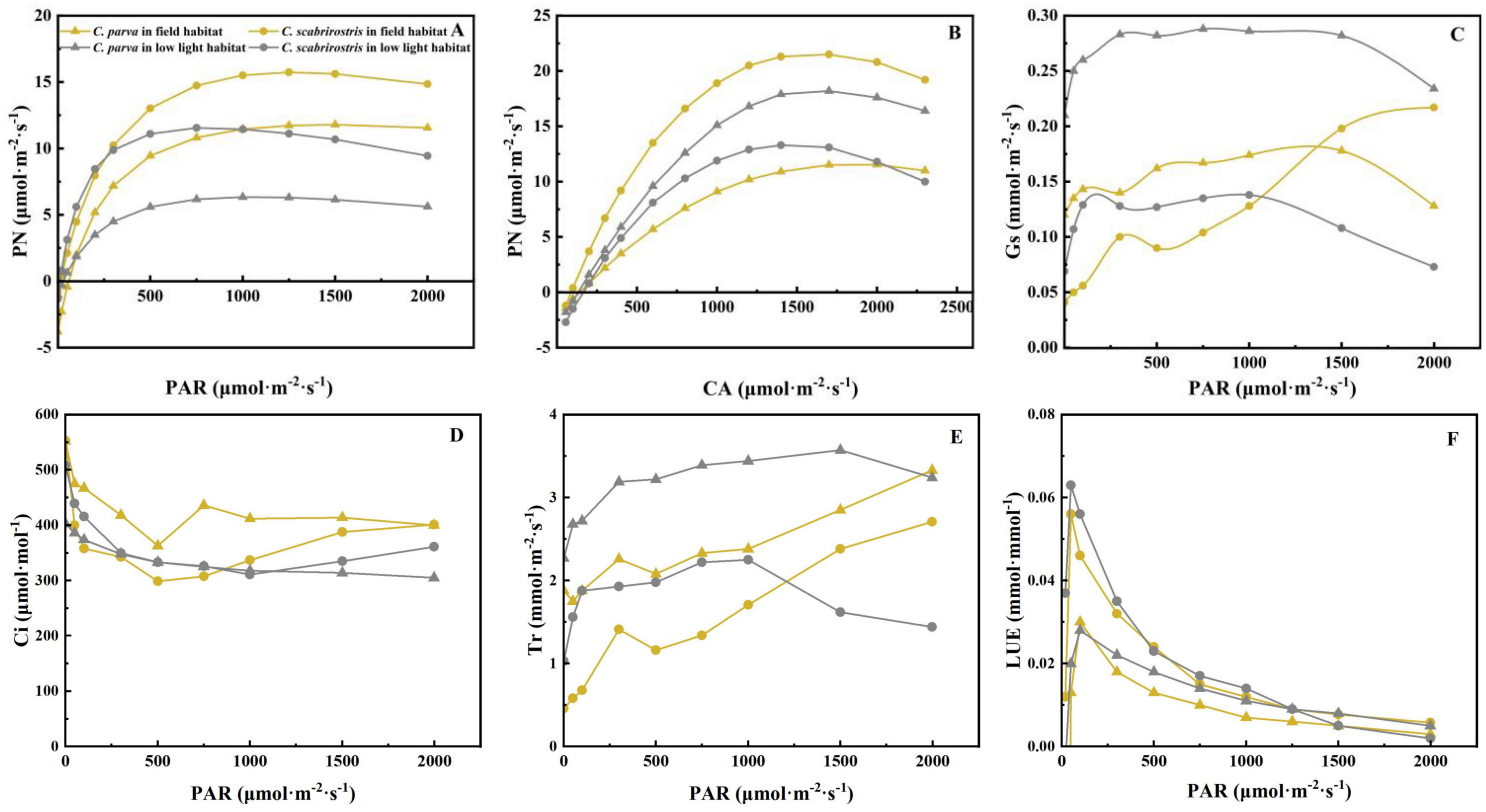
Light use efficiency of *C*. *parva* and *C*. *scabrirostris* with different photosynthetically active radiation. **(A)** Light response curves; **(B)** carbon dioxide response curves; **(C)** stomatal conductance; **(D)** transpiration rate; **(E)** intercellular CO_2_ concentration; **(F)** PN, net photosynthetic rate; PAR, photosynthetically active radiation; CA, air carbon dioxide concentration; Gs, Stomatal conductance; Tr, Transpiration rate; Ci, Intercellular CO_2_ concentration; LUE, light use efficiency.

The light response curves of *C. parva* and *C. scabrirostris* exhibited similar variations (**Figure 1A**). Overall, regardless of the environment, *C. scabrirostris* displayed greater photosynthetic capacity than *C. parva* (**Figure 1A**; **Table 1**). Under low-light conditions, the LSP of *C. parva* increased significantly, while that of *C. scabrirostris* decreased significantly (*p* < 0.05) (**Table 1**). Both *C. parva* and *C. scabrirostris* exhibited significant reductions in RD under low-light conditions. Compared to their natural habitats, both *Carex* species showed increased stomatal conductance (Gs) and transpiration rates (Tr) under low-light conditions (**Figures 1C, E**). However, in low-light conditions, the intercellular CO_2_ concentration (Ci) decreased in *C. parva*, while *C. scabrirostris* exhibited higher Ci levels at PAR < 800 (**Figure 1D**). Additionally, under PAR < 500, *C. scabrirostris* showed the highest light use efficiency (LUE) among the *Carex* species under low-light conditions (**Figure 1F**).

**Table 1:** Light response curve parameters, CO_2_ response curve parameters of *Carex parva* and *Carex scabrirostris*.

Specie		*Carex parva*		*Carex scabrirostris*	
Environments		Field habitat	Low-light habitat	Field habitat	Low-light habitat
Photo response parameters	α	0.02 ± 0.01c	0.04 ± 0.01bc	0.07 ± 0.01b	0.12 ± 0.03a
	Pn_max_	8.08 ± 0.82c	5.54 ± 1.97c	16.66 ± 2.23a	11.87 ± 2.33b
	LSP	1012.26 ± 25.31c	1212.67 ± 122.30b	1356.25 ± 3.63a	847.65 ± 41.29d
	LCP	54.32 ± 9.35a	32.92 ± 3.41b	14.66 ± 1.90c	11.31 ± 0.94c
	RD	3.95 ± 0.11a	1.12 ± 0.06b	1.12 ± 0.18b	0.67 ± 0.26c
CO_2_ response parameters	An_max_	14.46 ± 2.49bc	18.13 ± 0.70b	25.88 ± 3.38a	12.13 ± 1.60c
	CSP	1891.45 ± 34.60a	1808.10 ± 132.63a	1733.80 ± 122.80a	1440.34 ± 70.47b
	CCP	148.48 ± 3.56ab	130.75 ± 25.17c	99.78 ± 12.97b	176.11 ± 5.01a
	RP	3.95 ± 0.56a	4.66 ± 0.36a	2.24 ± 0.21b	4.02 ± 0.53a
	Vc_max_	36.33 ± 2.57a	30.61 ± 0.42b	36.47 ± 1.09ab	23.42 ± 2.10c
	J_max_	27.67 ± 2.52a	26.50 ± 2.02a	18.95 ± 1.84b	15.20 ± 0.53c

α, apparent quantum efficiency; Pn_max_, maximum net photosynthetic rate in light curve; LSP, light saturation point; LCP, light compensation point; RD, dark breathing rate; An_max_, maximum net photosynthetic rate in CO_2_ response curve; CSP, CO_2_ saturation point; CCP, CO_2_ compensation point; RP, photorespiration rate; VC_max_, maximum carboxylation rate; J_max_, maximum electron conductivity. Different letters following each value within a row indicate significant differences at p < 0.05. The same letter means no significant difference.

Additionally, akin to the changes observed in the light response curve, *C. scabrirostris* demonstrated a higher responsiveness to CO_2_ compared to *C. parva* (**Figure 1B**; **Table 1**). An_max_, CSP, VC_max_, and J_max_ showed significant reductions in *C. scabrirostris*, while CCP and RP exhibited significant increases. Conversely, in *C. parva*, CCP, and VCmax experienced significant decreases, while the other variables exhibited minimal or no response (**Table 1**).

### 3.2 Changes of plant morphology

**Figure 2:**
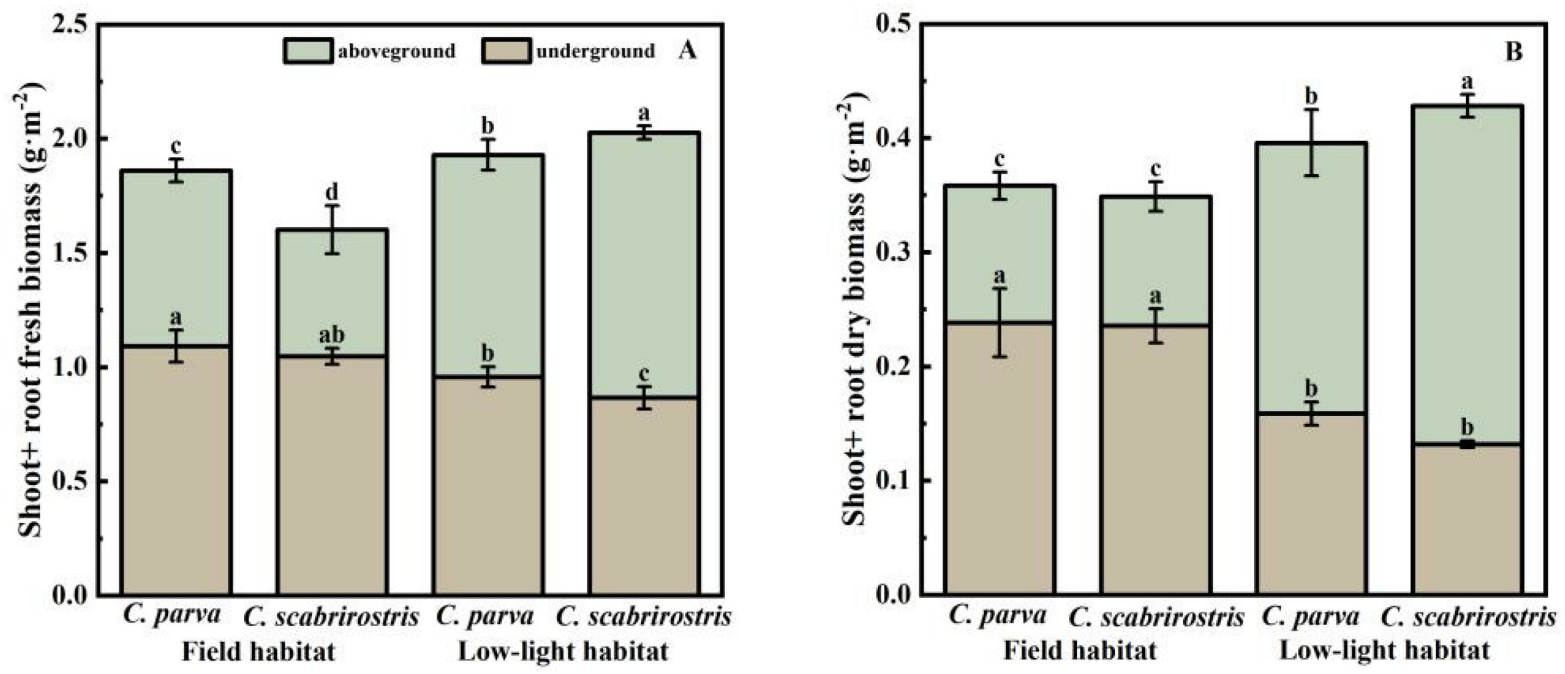
The effect of different environments on the aboveground and underground biomass changes in *C. parva* and *C. scabrirostris*. Different letters indicate significant differences in means between treatments based on ANOVA. Bars represent Means ± SE (standard errors). **(A)** is the aboveground and underground fresh biomass, **(B)** is the aboveground and underground dry biomass.

**Figure 3:**
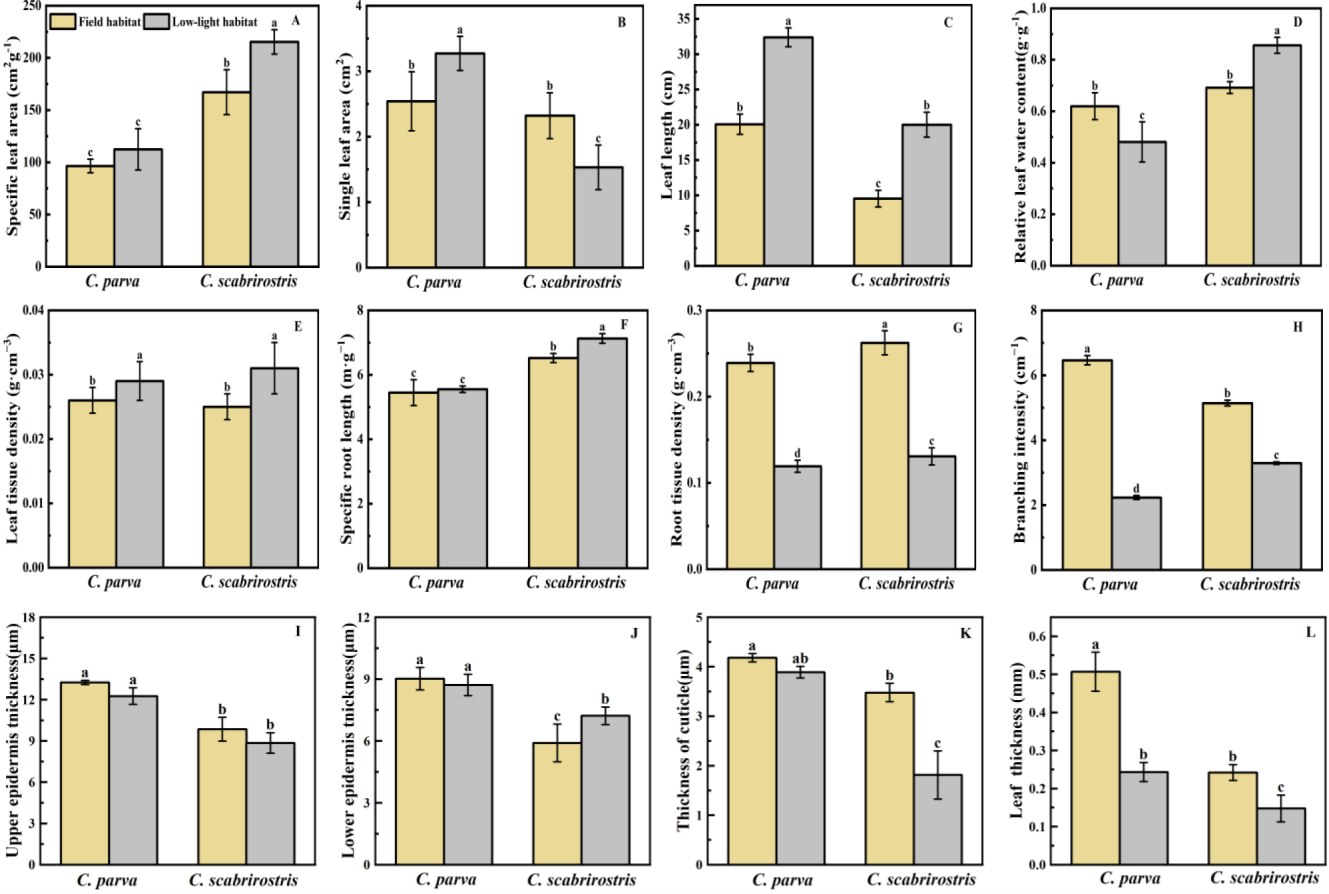
Effects of different environments on the morphological changes and leaf anatomical indices of *C. parva* and *C. scabrirostris*. Different letters indicate significant differences in means between treatments based on ANOVA. Bars represent Means ± SE (standard errors). **(A)** is the specific leaf area, **(B)** is the single leaf area, **(C)** is the leaf length, **(D)** is the relative leaf water content, **(E)** is the leaf tissue density, **(F)** is the specific root length, **(G)** is the root tissue density, **(H)** is the branching intensity, **(I)** is the upper epidermis thickness, **(J)** is the lower epidermis thickness, **(K)** is the thickness of cuticle, **(L)** is the leaf thickness.

Both *Carex* species significantly increased their aboveground biomass and decreased their belowground biomass under low-light conditions, leading to a reduced root-shoot ratio (**Figures 2A, B**). Morphologically, the leaves of both *Carex* species significantly elongate in response to low-light conditions (**Figure 3C**). In this setting, the leaf area of *C. parva* significantly increased, whereas the SLA remained relatively unchanged. Conversely, the leaf area of *C. scabrirostris* decreased significantly, accompanied by a notable increase in SLA (**Figures 3A, B**).

**Figure 4:**
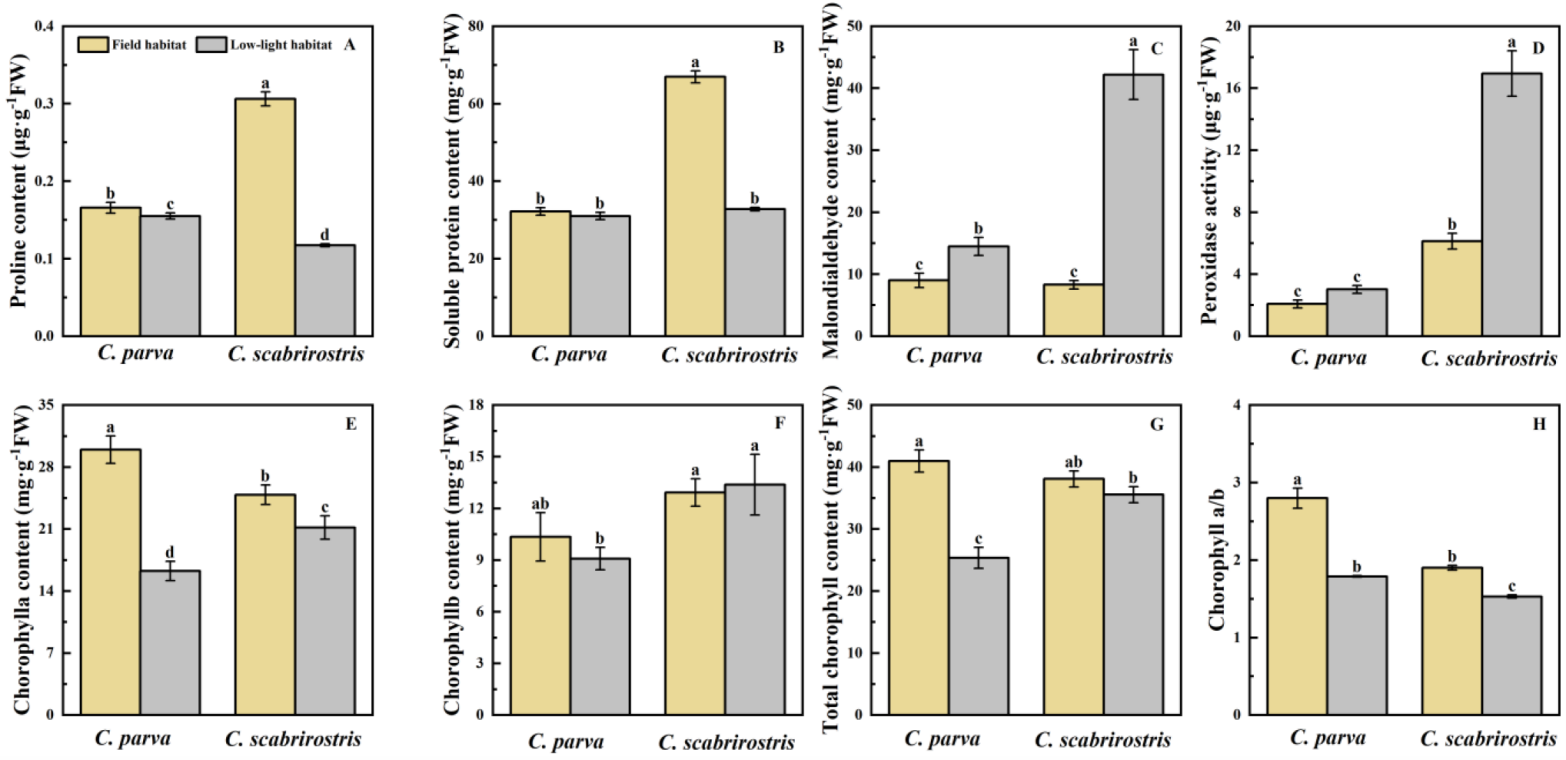
Effects of different environments on biochemical parameters of *C. parva* and *C. scabrirostris* leaves. Different letters indicate significant differences in means between treatments based on ANOVA. Bars represent Means ± SE (standard errors). **(A)** is the proline content, **(B)** is the soluble protein content, **(C)** is the malondialdehyde content, **(D)** is the peroxidase activity, **(E)** is the chorophylla content, **(F)** is the chorophyllb content, **(G)** is the total chorophyll content, **(H)** is the chorophya/b.

Concerning leaf relative water content (LRWC), the two *Carex* species exhibited distinct trends. LRWC decreased significantly in *C. parva*, whereas it significantly increased in *C. scabrirostris* (**Figure 3D**). Leaf tissue density (LTD) significantly increased in both *C. parva* and *C. scabrirostris* under low-light conditions (**Figure 3E**). **Figure 4** illustrates that under low-light conditions, the LET of *C. scabrirostris* increased significantly, while the CUT of both *Carex* species decreased significantly to varying degrees (*p* < 0.05). No significant changes were observed in the leaf dissection of *C. parva* (**Figures 4A–C**). Both *C. parva* and *C. scabrirostris* exhibited significantly thinner leaf thickness (LT) under low-light conditions (**Figure 4D**).

In terms of root morphology, the specific root length (SRL) of *C. scabrirostris* significantly increased under low-light conditions, while there was no significant change in *C. parva* (**Figure 3G**). However, both species of *Carex* showed a significant reduction in root tissue density (RTD) and branching intensity (BI) to varying degrees (**Figures 2F**, **3H**). It is worth noting that *C. scabrirostris* was found to have dauciform roots with an average density of 50.5 (number·g-1DW) in the field habitat, whereas the original dauciform roots disappeared after a period of growth in a low-light environment (**Table 2**).

**Table 2:** Dauciform root (DR) density, length and width of *Carex scabrirostris* in the field habitat.

Specie	DR density (number·g^−1^DW)	DR length (μm)	DR width (μm)
*Carex scabrirostris* (field habitat)	50.50	774.51	124.79

### 3.3 Changes of leaf biochemical parameters

The proline content in the leaves of both *Carex* species decreased significantly under low-light conditions (**Figure 4A**). SP levels significantly decreased in *C. scabrirostris*, whereas *C. parva* did not respond to SP (**Figure 4B**). Additionally, the MDA content in both *Carex* species significantly increased, with increases of 35.7% for *C. parva* and 80.9% for *C. scabrirostris* (**Figure 4C**). POD activity significantly increased by 62.5% in *C. scabrirostris*, (**Supplementary Table**), whereas no significant change was observed in *C. parva* (**Figure 4D**). Furthermore, under low-light conditions, both *Carex* species exhibited significant decreases in chlorophyll a and chlorophyll a/b (**Figures 4E, H**). Total chlorophyll content significantly decreased in *C. parva*, whereas no significant change was observed in *C. scabrirostris* (**Figure 4G**).

### 3.4 Plant plasticity, variability, and correlation between traits

**Figure 5:**
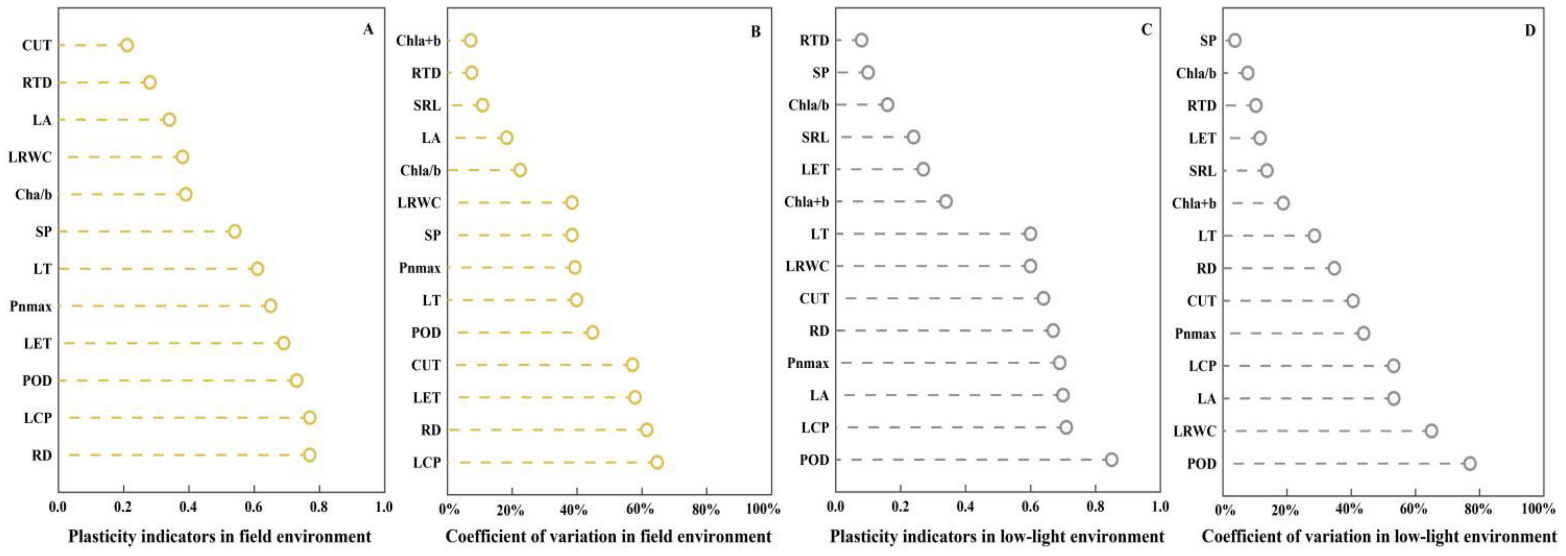
Plasticity index and coefficient of variation ranking of physiological and ecological indicators of *C. parva* and *C. scabrirostris* in different environments. LA, leaf area; LRWC, leaf relative water content; SRL, specific root length; RTD, root tissue density; CUT, cuticle thickness; LET, lower epidermal thickness; LT, leaf thickness; SP, soluble protein concentration; POD, peroxidase activity; Chla/b, chlorophyll a/b; Chla+b, total chlorophyll content; Pn_max_, maximum net photosynthetic rate in the light curve; LCP, light compensation point; RD, rate of dark respiration. **(A)** is the plasticity indicators in field environment, **(B)** is the coeffcient of variation index in field environment, **(C)** is the plasticity indicators in low-light environment, **(D)** is the coeffcient of variation index in low-light environment.

Plants in field habitats exhibit high sensitivity (PI ≥ 0.6) in terms of LT, net photosynthetic rate (Pn_max_), LET, POD, RD, and LCP response from the perspective of plant plasticity (**Figures 5A, C**). In terms of CUT, SRL, and chlorophyll a + b, they exhibit low sensitivity (PI < 0.2). In low-light environments, plants demonstrate high sensitivity (PI ≥ 0.6) in terms of LT, leaf dry matter content (LDWC), CUT, RD, Pn_max_, leaf area (LA), LCP, and POD. However, they exhibit low sensitivity (PI < 0.2) in terms of RTD, SP, and chlorophyll a/b.

From the perspective of plant variability (**Figures 5B, D**), plants in field habitats exhibit greater variability (CV > 50%) in terms of CUT, LET, RD, and LCP. In terms of LA, SRL, RTD, and chlorophyll a + b, they demonstrate smaller variability (CV <20%). However, in low-light environments, plants demonstrate greater variability (CV >50%) in terms of POD, LDWC, LA, and LCP. On the other hand, they show smaller variability (CV <20%) in terms of SP, chlorophyll a/b, SRL, LET, chlorophyll a + b, and RTD. The ranking of plant plasticity and variability remains consistent across different environments.

**Figure 6:**
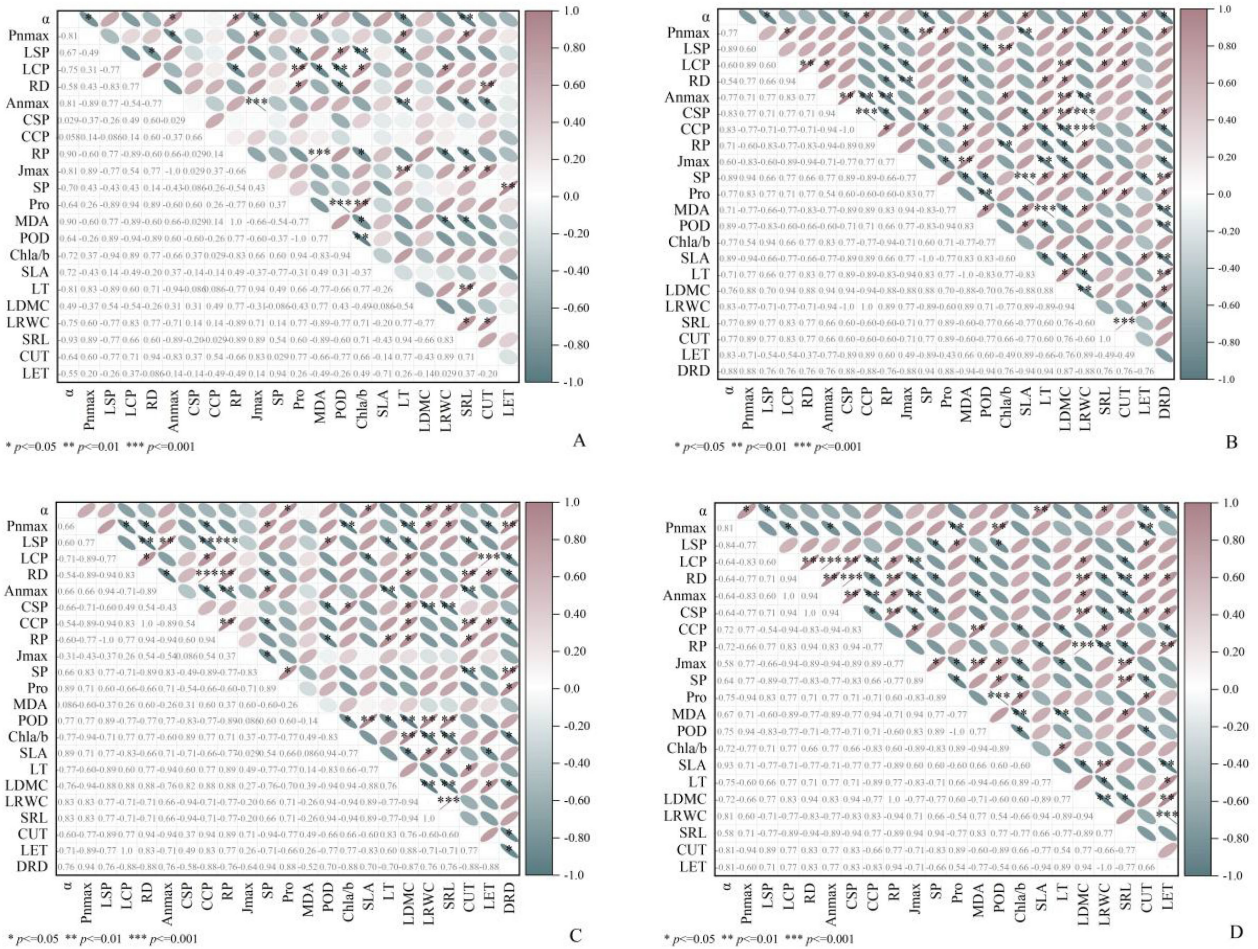
Spearman correlation analysis showed the relationship between functional traits of *C. parva* and *C. scabrirostris*. **(A)** is the relationship between the functional traits of *C. parva*; **(B)** is the relationship between the functional traits of *C. scabrirostris*; **(C)** is the relationship between the functional traits of two *Carex* species in the field environment; **(D)** is the relationship between the functional traits of two *Carex* species in the low-light environment. In the area in the lower left corner, the number represents the correlation coefficient. In the upper right corner, blue indicates a negative correlation, red indicates a positive correlation, and * indicates a significant correlation. α, apparent quantum efficiency; Pn_max_, maximum net photosynthetic rate in the light curve; LSP, light saturation point; LCP, light compensation point; RD, rate of dark respiration; An_max_, maximum net photosynthetic rate (CO_2_—response curves); CSP, CO_2_ saturation point; CCP, CO_2_ compensation point; RP, photorespiration rate; J_max_ maximum electron conductivity; SP, soluble protein; Pro, proline content; MDA, malondialdehyde content; POD, peroxidase activity; Chla/b, Chlorophyll a/b; SLA, specific leaf area; LT, leaf thickness; LDWC, leaf dry weight/fresh weight; LRWC leaf relative water content; SRL, specific root length; CUT, cuticle thickness; LET, lower epidermal thickness.

In terms of morphological indicators, morphologically, SLA shows a positive correlation with LRWC and a negative correlation with LET, irrespective of the environment (**Figures 4D**, **6C**). Regarding intraspecific correlations of *C. scabrirostris* across different environments, dauciform root density (DRD) shows significant positive correlations with Pn_max_ and SP, and significant negative correlations with VC_max_, J_max_, POD, and SLA (**Figure 6B**). In the natural habitat promoting dauciform root growth, the indicators that are significantly positively correlated with DRD remain consistent, while they show significant negative correlations with LCP, RD, chlorophyll a/b, CUT, and LET (**Figure 6C**). Regarding photosynthetic parameters, in natural habitats, Pn_max_ exhibits positive correlations with SRL, DRD, and SP, while significantly negatively correlated with LDMC and chlorophyll a/b (**Figure 6C**). In low-light environments, Pn_max_ shows a significant positive correlation with POD (**Figure 6D**). Regarding physiological characteristics, in natural habitats, POD exhibits significant positive correlations with SLA and LRWC, while significantly negatively correlated with LDMC and chlorophyll a/b. In low-light environments, POD shows a negative correlation with chlorophyll a/b.

### 3.5 Redundancy analysis

**Figure 7:**
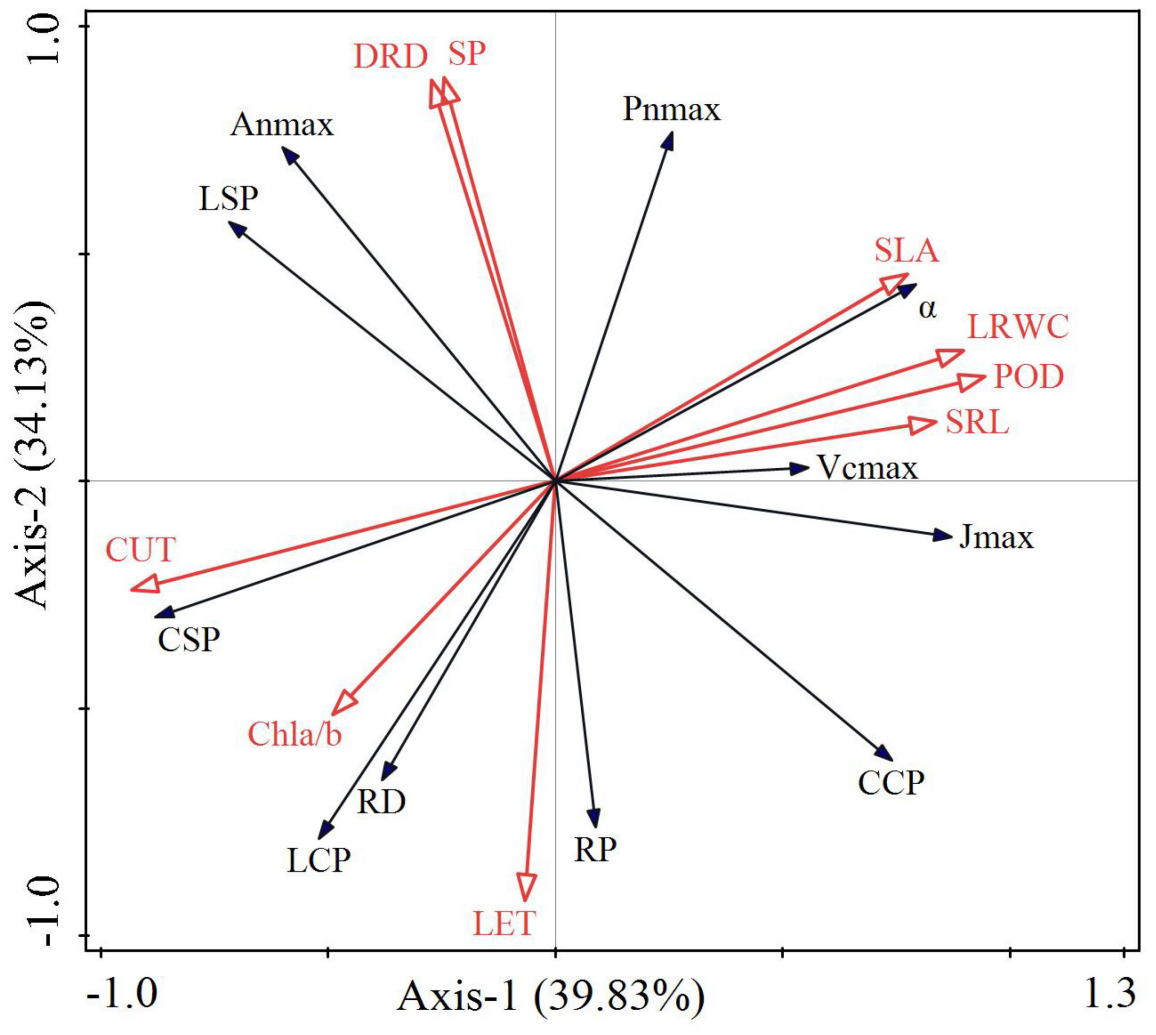
RDA analysis between photosynthetic and ecophysiological properties of two Carex species. α, apparent quantum efficiency; Pn_max_, maximum net photosynthetic rate in light curve; LSP, light saturation point; LCP, light compensation point; RD, dark respiration rate; An_max_, maximum net photosynthetic rate in CO_2_ response curve; CSP, CO_2_ saturation point; CCP, CO_2_ compensation point; RP, photorespiration rate; VC_max_, maximum carboxylation rate; J_max_, maximum electronic conductivity; POD, peroxidase activity; DRD, dauciform root density; SLA, specific leaf area; LRWC, leaf relative water content; Chla/b, chlorophylla/b; SP, soluble protein; LET, lower epidermal thickness; CUT, cuticle thickness; SRL, specific root length.

The explanatory variables of the first and second axes were 39.83% and 34.13%, respectively, indicating that the first and second axes accounted for 73.96% of the variation in the photosynthetic characteristics of the two *Carex* species (**Figure 7**). Among the explanatory variables, POD had the longest arrow and the largest projected area, explaining 37.8% of the variation with a significant *p*-value of 0.002. This indicates a strong correlation with photosynthetic characteristics, significantly affecting them (*p* < 0.05). Additionally, POD had the highest contribution value of 39.2% and showed a positive correlation (acute angle) with α and Pn_max_, while exhibiting a negative correlation (obtuse angle) with LCP and RD. DRD explained 31.10% of the variation with a *p*-value of 0.010, significantly affecting photosynthetic characteristics, positively correlating with Pn_max_, An_max_, and LSP, and negatively correlating with RP. SLA accounted for 15.5% of the variation with a *p*-value of 0.004, significantly affecting photosynthetic characteristics and positively correlating with Pn_max_ and J_max_. Among these variables, SLA and α showed the smallest angle. LRWC explained 3.80% of the variation with a *p*-value of 0.042, significantly affecting photosynthetic characteristics, positively correlating with α and Vc_max_, and negatively correlating with CSP. The results of RDA were consistent with the correlation values obtained from Pearson analysis.

## 4 Discussion

### 4.1 Key factors affecting the photosynthetic capacity of two *Carex* species

In this experiment, immediately after sampling the field habitat plants, we simulated the native environments of *C. parva* and *C. scabrirostris* in an artificial climate chamber in order to obtain plants acclimated in low-light conditions. By controlling environmental factors, we minimized phenological differences among the two groups (Rosbakh et al., 2021) and reduced growth differences to the lowest possible level. We used the same soil substrate as in their field habitats, maintained humidity and soil moisture according to their native conditions, and strictly regulated the diurnal temperature variations in the climate chamber. This approach ensured that light intensity was the major variable, allowing us to investigate the acclimation changes and key factors influencing the photosynthetic capacity of the two *Carex* species under low-light conditions.

This study found that the POD activity, SLA, DRD, and LRWC of the two Carex species contributed 88.2% to their photosynthetic capacity (**Table 3**) and were significantly correlated with photosynthesisrelated indicators (*p* < 0.05). Among these, POD activity made the largest contribution to the photosynthesis-related indicators of the two *Carex* species. Under low-light stress conditions, the two *Carex* species produce reactive oxygen species that damage chloroplasts, resulting in decreased photosynthetic capacity. In order to maintain the redox balance and preserve the photosynthetic function of chloroplasts, both *Carex* species exhibited high POD activity to eliminate reactive oxygen species, stabilizing cell membranes and the photosystem (Liang et al., 2009; Zhang et al., 2022). Further analysis reveals that the plasticity and variability of POD activity showed strong regulatory potential under different environmental conditions, reinforcing the role of POD as a key factor in the response of plants to environmental variation. Therefore, they are key indicators influencing the habitat adaptability of *Carex* species. This also confirms that POD will change accordingly with environmental changes to help plants cope with adverse environments. Shading increased the POD activity of *Cedrela fissilis*, consistent with the findings of this study (Barbosa et al., 2022).

**Table 3:** Contribution value of each index to photosynthesis.

Name	Explains %	Contribution %	pseudo-*F*	P
POD	37.8	39.2	6.1	0.002
DRD	31.1	32.2	9.0	0.010
SLA	15.5	16.0	7.9	0.004
LRWC	3.8	4.0	2.3	0.042
Chla/b	2.6	2.7	1.7	0.086
LET	1.7	1.8	1.2	0.458
SP	1.5	1.5	1.0	0.642
CUT	1.2	1.3	0.8	0.130
SRL	1.3	1.3	0.8	0.602

POD, peroxidase activity; DRD, dauciform root density; SLA, specific leaf area; LRWC, leaf relative water content; Chla/b, chlorophylla/b; SP, soluble protein; LET, lower epidermal thickness; CUT, cuticle thickness; SRL, specific root length; F: square of dispersion/degree of freedom within and between groups; p, p-value.

SLA and LRWC are essential indicators of leaf structure and morphology in plants. SLA is closely related to plant growth and survival strategies, associated with light capture and photosynthetic capacity. A larger leaf area facilitates capturing more light energy and enables faster plant growth potential. Plants with effective photosynthesis maintain high LRWC, preserving chloroplast structure and function. Both *Carex* species exhibited low SLA in fully illuminated environments, indicating that excessive investment in leaf morphology for efficient light absorption was unnecessary. In low-light conditions, *C. scabrirostris* actively increases its leaf area ratio to maximize light capture, maintaining relatively high LRWC, effectively preserving chloroplast structure and photosystem II function, enabling efficient photosynthesis. In this environment, *C. parva* showed a non-significant increase in SLA and a decrease in LRWC. This may be due to *C. parva*’s weaker ability to adapt to low light or the different ways in which the leaf morphology of the two *Carex* species adapts to light intensity. Meanwhile, both SLA and LRWC showed strong plasticity and variability under low light, serving as sensitive indicators of habitat response. This is also supporting evidence that the two species of *Carex* adapted to different light environments by regulating these indicators. In summary, plants adapt to different light environments by adjusting the morphological structure and physiological changes in their leaves.

As not all *Care*x species produce dauciform roots, this study finds that POD activity and LRWC as the most crucial physiological indicators affecting the photosynthetic capacity of the two *Carex* species during the transition from full light to low-light environments. Additionally, SLA is considered one of the most important indicators from a morphological perspective affecting the photosynthetic characteristics of these two *Carex* species. Valladares et al. (2006) considers the PI and CV to be relatively simple and effective methods, noting a strong correlation between each method. In this study, the rankings of the PI and CV are generally consistent, reflecting the sensitivity of each index to habitat responses. In summary, POD significantly influences the photosynthetic characteristics of both *Carex*. Under low-light conditions, the POD, SLA, and LRWC of *C. scabrirostris* are all significantly greater than those of *C. parva*, indicating that *C. scabrirostris* possesses higher photosynthetic efficiency and greater light energy utilization.

### 4.2 Responses of two *Carex* species to low-light environment

In this experiment, both *C. parva* and *C. scabrirostris* showed increased stomatal conductance, transpiration rate, and aboveground dry matter content under low-light conditions (PAR = 150). These results suggest that in such an environment, the water vapor exchange between the leaves of both *Carex* species and the external environment is promoted, thereby enabling the accumulation of photosynthetic products. *C. parva* reduces its LCP under low-light conditions but accumulates organic matter by increasing its LSP. Under low-light conditions, both the Pn_max_ and LSP of *C. scabrirostris* significantly decreased, indicating a reduction in its photosynthetic capacity. This reduction, in turn, enhances its adaptability to low-light environments (Fu et al., 2017). The Pn_max_ of both *Carex* species under low-light conditions is lower than in the field environment, possibly indicating acclimation to low light. Furthermore, regardless of the light environment, Pn_max_ of *C. scabrirostris* is significantly higher than that of *C. parva*, demonstrating its superior photosynthetic ability. Further analysis of LUE indicates that *C. scabrirostris* exhibits the highest LUE under low-light conditions, suggesting strong adaptability and survival capabilities in such environments. Both Vc_max_ and J_max_ limit plant photosynthesis, and under low-light conditions compared to their natural environments, both *Carex* species exhibited varying degrees of reduction in Vc_max_ and J_max_. This could be one of the reasons for the weakened photosynthetic capacity, consistent with the findings of this study (Choi et al., 2021).

Plants have been shown to adapt to low-light conditions by increasing their chlorophyll content and reducing the a/b ratio under shaded conditions (Yao et al., 2016; Hirano et al., 2019). In low-light environments, the chlorophyll a/b ratio significantly decreases in both *Carex* species. *C. scabrirostris* displayed no significant alterations in total chlorophyll content, while *C. parva* significantly decreased. This also confirms that *C. scabrirostris* is more shade tolerant. Additionally, the SP content of both *Carex* species is positively correlated with Pn_max_ and An_max_. Pn_max_ and An_max_ decrease significantly under low-light conditions. This indicates that photosynthesis in both *Carex* species is hindered compared to their native habitat in such low-light conditions, potentially reducing the photosynthetic yield of plants, thus limiting their ability to produce more proteins (Miao et al., 2023). This finding is important for understanding the growth strategies and physiological mechanisms of Carex species in low-light environments. *C. scabrirostris* exhibits the highest levels of SP and Pn_max_ in its habitat, providing nutrients for plant growth and aiding in better acclimation to the environment (Wang et al., 2021). Additionally, the excessive accumulation of lipid peroxidation products, measured as MDA, in both *Carex* species under low-light environments can cause damage to chloroplasts through the generation of reactive oxygen species, leading to a decline in plant photosynthetic capacity. To maintain internal redox homeostasis and sustain chloroplast function, both *Carex* species demonstrate higher POD activity to scavenge reactive oxygen species and maintain intracellular redox balance, thus keeping the cell membrane and the photosystem stable (Smirnoff and Arnaud, 2019).

Plants can maximize their photosynthetic efficiency and capacity by adjusting leaf area, and the increase in leaf area determines the light interception capacity (Weraduwage et al., 2015; Yao et al., 2016). Under full sunlight, two species of *Carex* actively regulate leaf area and aboveground biomass, exhibiting the lowest SLA and aboveground biomass. This acclimation avoids excessive light absorption and inhibition (Yao et al., 2016). In low-light environments, *C. parva* increases its individual leaf area to obtain more light energy. On the contrary, leaf thickness is significantly negatively correlated with individual leaf area. These morphological changes can optimize the leaf’s ability to capture light and alleviate potential light inhibition effects in *C. parva*. Leaf thickness and SLA of *C. scabrirostris* show a similar trend to *C. parva*. However, the individual leaf area of *C. scabrirostris* decreases significantly, although this decrease may be indicative of the plant’s capability to transport resources from aboveground to underground. This may explain why *C. scabrirostris* has decreased underground biomass while increasing aboveground biomass. The biomass of aboveground organisms is inversely proportional to leaf area, further indicating that the allocation of aboveground and belowground biomass is influenced by leaf area. Upper epidermal cell thickness in two *Carex* species is found to display a significant negative correlation with SLA while demonstrating a noteworthy positive correlation with the chlorophyll a/b ratio. Thinning of the epidermis thickness of two *Carex* species increases the light-exposed surface area, enhancing the light capture capacity of the leaves. It facilitates light penetration through the leaf surface, promotes photochemical reactions within leaf cells, and ultimately improves photosynthetic efficiency (Rôças et al., 2001).

In low-light environments, the RTD and BI of both *C. parva* and *C. scabrirostris* are significantly reduced, which decreases their competitiveness in the underground (Cheng et al., 2009). The SRL of *C. scabrirostris* significantly increases in low-light environments, indicating an active enhancement of root absorption for water and nutrients, thereby improving its adaptability to low-light conditions (Bordron et al., 2021). Consequently, *C. scabrirostris* exhibits a high level of competitiveness in terms of nutrient and water resources, promoting rapid growth even in low-light conditions (Birouste et al., 2014).

### 4.3 Growth strategies of two *Carex* species in two different environments

In addressing the third research question, it is possible to answer from both the perspective of trait variation, plasticity, and the leaf economic spectrum. Analyzing CV and PI is necessary to accurately reflect a species’ response to environmental changes and resource competition during community assembly (Donovan et al., 2011; Navarro and Hidalgo-Triana, 2021). Compared with roots, leaves exhibit greater plasticity and variability (**Figure 4**). This suggests that the root systems of the two *Carex* species remain relatively stable in heterogeneous environments. Meanwhile, plants trade-off various traits and make corresponding changes with environmental variations (Römermann et al., 2016). The different sensitivities of morphology, leaf anatomical structures, and photosynthetic parameters in this study to environmental changes indicate that plants also trade-off among different morphologies, leaf anatomical structures, and photosynthetic capacities to achieve optimal survival strategies, which benefit individual survival and population development. On the other hand, plants face the combined influences of various habitat factors, and a positive response of a certain trait to one environmental factor may be a negative response to another environmental factor (Langley et al., 2022). Plants also balance their responses to different habitat factors. In low-light environments, light is the main environmental factor limiting the growth of both *Carex* species. After weighing the pros and cons, both *Carex* species prioritize increasing leaf area and accumulating aboveground biomass to cope with the stressful conditions of low light. In field habitats, the CV values of LCP, LET, CUT, and RD are all above 50%, indicating that plants maintain internal leaf moisture through thicker cuticles and epidermal layers (Guo et al., 2023) and increase their photosynthetic capacity with higher LCP and faster RDs. Under low-light conditions, the CV values of LA, LRWC, and POD are all above 50%, which is related to the reduction in photosynthetic capacity. Plants strategically increase leaf area and utilize POD to eliminate reactive oxygen species, thereby optimizing light absorption and addressing limitations under low-light conditions.

Theoretical analysis of leaf economics spectra reveals that the investment strategies of both *Carex* species remain unchanged, whether in field habitats or low-light environments. *C. parva*, with weaker photosynthetic ability, a smaller SLA, and higher leaf dry matter accumulation, is categorized as a “slow-investment-high-return” species. In contrast, *C. scabrirostris* is a “fast-investment-low-return” species due to its larger SLA, higher photosynthetic capacity, and lower leaf dry matter accumulation (Stearns, 1998). *C. scabrirostris* employs a rapid investment strategy, enabling it to quickly occupy space and resources, though it may be less stable in long-term competition compared to species with slower investment strategies (Wright and Grier, 2012). Notably, *C. scabrirostris* shows significant variation in its investment strategy depending on the environment. In natural habitats, it allocates more resources underground, enhancing root expansion and nutrient absorption to address nutrient limitations. This strategy likely reduces leaf maintenance costs and increases resource-use efficiency, helping the species cope with the potential stress of high light intensity. Conversely, under low-light conditions, *C. scabrirostris* prioritizes aboveground growth, such as leaf development and optimization of photosynthetic structures, to maximize the capture of limited light resources. This flexible investment strategy underscores *C. scabrirostris*’s high adaptability to environmental changes and the diversity of its survival strategies. *C. parva* maintains consistent investment strategies in both environments, possibly due to its slower investment strategy or lack of response within a short time frame. Through in-depth analysis, this study further reveals that these species employ different response strategies in their aboveground parts when facing low-light conditions. *C. parva* enhances light capture efficiency by increasing leaf area, while *C. scabrirostris* reduces individual leaf area and leaf mass but significantly increases aboveground biomass, potentially due to an increased number of blades. This illustrates the resource allocation and compromise strategies employed by plant species in environments with limited resources, aligning with their trait adaptations and functional demands (Westoby and Wright, 2006; Heberling and Fridley, 2012). Under low-light conditions, *C. scabrirostris* allocates more resources to aboveground growth, altering its growth strategy, while *C. parva* remains unchanged in this experiment. These different responses reflect the long-term coexistence strategies of the two *Carex* species in relation to environmental conditions and resource competition.

From the perspective of the root economics spectrum, the two *Carex* species adopted a rapid investment strategy in a simulated urban low-light environment, whereas a conservative investment strategy was observed in their natural habitat (Martín-Robles et al., 2019). This finding is consistent with the conclusions drawn from the leaf economics spectrum. Notably, under simulated urban low-light conditions, *C. scabrirostris* was unable to produce dauciform roots. RDA and correlation analysis revealed a significant association between the presence of dauciform roots and the plant’s photosynthetic capacity. In low-light conditions, although the investment strategy for roots and leaves is rapid investment, *C. scabrirostris* prioritizes resources to the leaves to optimize light capture and improve photosynthetic efficiency. As a result, this leads to reduced investment in the belowground components, including the formation and maintenance of dauciform roots. Consequently, after growing in the same soil environment for a period of time, the dauciform roots of *C. scabrirostris* gradually disappeared.

## 5 Conclusion

This experiment significantly advanced our understanding of the response mechanisms of *C. parva* and *C. scabrirostris* to low-light conditions. First, indicators such as POD activity, SLA, and relative water content significantly influenced the photosynthetic capacity of the two *Carex* species. Secondly, under low-light conditions, *C. parva* exhibited a slow investment-return response strategy, while *C. scabrirostris* adopted a fast investment-return response strategy. Both *Carex scabrirostris* and *Carex parva* allocate more resources to aboveground growth in low-light environments to better adapt to the reduced light conditions. Third, in the simulated urban low-light environment, neither *C. parva* nor *C. scabrirostris* produced dauciform roots. The study found a strong correlation between dauciform root formation in *Carex* species and light intensity. Within the scope and conditions of these experiments, POD activity emerged as a key player in maintaining plant growth and photosynthetic capacity under low-light conditions. Delving deeper into the regulatory mechanisms of POD to light will provide valuable insights for optimizing plant light-use efficiency and enhancing adaptability to stressful environments.

Furthermore, both species showcased superior shade tolerance under simulated low-light urban environments, particularly *C. scabrirostris*. These findings hold promise for their potential as excellent turfgrass varieties for low-light environments in cities, laying a solid research foundation for their future cultivation and domestication. To safeguard species diversity in lawn grass and bolster the stability of urban lawns, this study advocates for further research focusing on the developmental potential of *Carex* species and their tolerance to high shade conditions.

## Data Availability

The raw data supporting the conclusions of this article will be made available by the authors, without undue reservation.
