# Supplementary material for: Carex parva and Carex scabrirostris adopt diverse response strategies to adapt to low-light conditions
Source: Front Plant Sci. 2024 Oct 14;15:1432539. doi: 10.3389/fpls.2024.1432539 (PMC11513331; doi:10.3389/fpls.2024.1432539)
Supplement: Supplementary file 1 [file Table1.docx]

Supplementary table: aboveground and belowground biomass indexes, leaf and root morphological indexes, leaf anatomical indexes, and physiological indexes of *Carex parva* and *Carex scabrirostris.*

| Specie | | *Carex parva* | | *Carex scabrirostris* | |
| --- | --- | --- | --- | --- | --- |
| environments | | Field habitat | Low-light habitat | Field habitat | Low-light habitat |
| Aboveground and belowground biomass indexes | Shoot fresh biomass | 0.77±0.05c | 0.97±0.07b | 0.55±0.10d | 1.16±0.03a |
|  | Root dry biomass | 1.09±0.07a | 0.96±0.04b | 1.05±0.04ab | 0.87±0.05c |
|  | Shoot dry biomass | 0.12±0.01c | 0.24±0.03b | 0.11±0.01c | 0.30±0.01a |
|  | Root fresh biomass | 0.24±0.03a | 0.16±0.01b | 0.24±0.02a | 0.16±0.003b |
| Plants morphological indexes and leaf anatomical indexes | Specific leaf area | 96.40±6.52c | 112.42±19.79c | 167.07±21.47b | 215.26±11.67a |
|  | Single leaf area | 2.54±0.45b | 3.27±0.26a | 2.32±0.35b | 1.53±0.34c |
|  | Leaf length | 20.07±1.43b | 32.4±1.34a | 9.53±1.18d | 20.03±1.76b |
|  | Relative leaf water content | 0.62±0.05b | 0.48±0.08c | 0.69±0.02bb | 0.86±0.03a |
|  | Leaf tissue density | 0.026±0.002b | 0.030±0.003a | 0.025±0.002b | 0.031±0.004a |
|  | Specific root length | 5.45±0.40c | 5.55±0.10c | 6.52±0.14b | 7.13±0.15a |
|  | Root tissue density | 0.23±0.01b | 0.12±0.01d | 0.26±0.01a | 0.13±0.01c |
|  | Branching intensity | 6.46±0.14a | 2.23±0.06d | 5.14±0.09b | 3.29±0.04c |
|  | Leaf thickness | 0.51±0.05a | 0.24±0.03b | 0.24±0.02b | 0.15±0.04c |
|  | Upper epidemis tnickness | 13.25±0.15a | 12.26±0.60a | 9.85±0.86b | 8.86±0.75b |
|  | Lower epidemis tnickness | 9.02±0.54a | 8.71±0.52a | 5.90±0.92c | 7.22±0.43b |
|  | Thickness of cuticle | 4.18±0.09a | 3.89±0.18ab | 3.48±0.19b | 1.81±0.49c |

Continued: aboveground and belowground biomass indexes, leaf and root morphological indexes, leaf anatomical indexes, and physiological indexes of *Carex parva* and *Carex scabrirostris.*

| Specie | | *Carex parva* | | *Carex scabrirostris* | |
| --- | --- | --- | --- | --- | --- |
| environments | | Field habitat | Low-light habitat | Field habitat | Low-light habitat |
| Leaf anatomical indexes, | Proline | 0.17±0.01b | 0.15±0.004c | 0.31±0.01a | 0.12±0.003d |
|  | Soluble protein | 32.20±0.99b | 31.01±0.96b | 66.93±1.57a | 32.82±0.44b |
|  | Malondialdehyde content | 9.01±1.14c | 14.47±1.45b | 8.30±0.67c | 42.20±4.01a |
|  | Peroxidase activity | 2.08±0.25c | 3.02±0.25c | 6.12±0.50b | 16.94±1.46a |
|  | Chorophylla content | 29.97±1.55a | 16.27±1.06d | 24.88±1.09b | 21.18±1.33c |
|  | Chorophyllb content | 10.35±1.40ab | 9.05±0.65b | 12.93±0.80a | 13.39±1.76a |
|  | Total chorophyll content | 40.99±1.80a | 25.36±1.70c | 38.1±1.30ab | 35.56±1.33b |
|  | Chorophyll a/b | 2.80±0.13a | 1.79±0.01b | 1.90±0.03b | 1.53±0.02c |

Different letters following each value within a row indicate significant differences at *p*<0.05. The same letter means no significant difference.
